# Person-centered care in ophthalmology: current knowledge and perspectives

**DOI:** 10.3325/cmj.2024.65.156

**Published:** 2024-04

**Authors:** Snježana Kaštelan, Neda Pjevač, Marijana Braš, Veljko Đorđević, Nada Pjevač Keleminić, Juan E. Mezzich

**Affiliations:** 1 Department of Ophthalmology, Dubrava University Hospital, University of Zagreb, School of Medicine, Zagreb, Croatia *snjezanakastelan@yahoo.com*; 2 Department of Medical Statistics, Epidemiology and Medical Informatics, University of Zagreb, School of Medicine, Andrija Štampar School of Public Health, Zagreb, Croatia; 3 Center for Palliative Medicine, Medical Ethics and Communication Skills, University of Zagreb School of Medicine, Zagreb, Croatia; 4 Department of Family Medicine, University of Zagreb, School of Medicine, Zagreb, Croatia; 5 Health Centre Zagreb-Centar, Zagreb, Croatia; 6 Icahn School of Medicine at Mount Sinai, New York, NY; 7 Hipolito Unanue Chair of Person Center Medicine, San Marcos National University, Lima, Peru; 8 International College of Person-Centered Medicine

Person-centered medicine prioritizes holistic care by placing the individual at the center of health care practices. It emphasizes addressing all aspects of well-being, including biological, psychological, sociological, cultural, spiritual, and environmental factors (1,2).

Mezzich et al (3-5), representing the International Network for Person-centered Medicine (now the International College of Person-centered Medicine), have outlined crucial values of person-centered care. These include a comprehensive theoretical framework, addressing both illness and positive health, prioritizing person-centered research, upholding independence, accountability, and dignity, and promoting partnerships (3-5). Andrija Štampar was another influential advocate for people-centered care, emphasizing patient needs and well-being. His contributions gained prominence in the 20th century, alongside advances in evidence-based medicine primarily focused on organs and illnesses (6).

Human-centered medicine extends beyond psychosomatic medicine by prioritizing social relationships. This involves fostering interactions between physicians and patients and promoting collaboration between medical professionals and the patient's family (1,7). It is crucial to understand individuals within their challenges and empower them to perceive health as a lifelong journey with continuous support. Physicians should see themselves not just as health guardians but as holistic individuals with scientific expertise and ethical commitments, practicing medicine “from the person's perspective.” Additionally, there is a focus on practicing medicine “with the person,” emphasizing collaboration with individuals seeking assistance (1,7).

The concept of human-centered medicine traces back to ancient civilizations, including the Chinese, Indians, and renowned figures such as Hippocrates and Aristotle, all advocating personalized health approaches. However, scientific progress in the past century has redirected medicine's focus from individuals to diseases. Presently, there is a growing aspiration to restore medicine's fundamental objectives, which have been overshadowed by political and pharmaceutical influences. Patient-centered clinical care is pivotal in global initiatives to prioritize individuals and their unique contexts across diverse medical disciplines, aiming to place them at the center of health care services (1,7-9).

This article aims to elucidate post-diagnostic care models for common ophthalmological conditions with significant impacts on vision. We analyzed the structural and procedural aspects of care delivery, examining outcomes for both health systems and patients, and exploring knowledge gaps and limitations associated with achieving patient-centered care. Therefore, our goal is to review the various patient-centered care methods used in the treatment of ophthalmic diseases.

## Person-centered care in ophthalmology

Currently, there is no standardized approach for delivering patient-centered care, both in a general health care context and specifically within the field of ophthalmology (1-3).

In ophthalmology, special emphasis is placed on understanding the pathology, clinical aspects, and therapeutic interventions for various eye diseases, with a particular focus on addressing refractive anomalies. Prevalent chronic eye conditions contributing to vision impairment include diabetic retinopathy, glaucoma, macular degeneration, and cataracts. Worldwide, glaucoma, macular degeneration, and diabetic retinopathy are the main contributors to irreversible vision loss, while cataracts remain the primary cause of reversible visual impairment, effectively treatable with surgical intervention (10,11).

In many cases, patients often rely on their ophthalmologists for effective disease management, frequently with limited personal involvement or understanding of their care. However, patients often assess the quality of health care not only based on disease outcome measures but also on interpersonal relationships and communication with their health care providers (10,12). This points to an inconsistency between the care provided by a majority of ophthalmologists and patients’ expectations. Patients with chronic eye conditions need regular examinations and follow-up care (12,13). Currently, ophthalmology consultations predominantly focus on improving clinical aspects of disease, namely intraocular pressure (IOP), and controlling the progression of visual field damage in glaucoma. Furthermore, in exudative age-related macular degeneration, attention is directed toward treating macular hemorrhage or reducing the macular thickness (14,15).

Over the past decade, the focus of cataract surgery has evolved from addressing solely the functional symptoms associated with cataracts to enhancing the refractive results of the procedure, which includes the correction of refractive error (16,17). A notable advancement in person-centered patient care in ophthalmology is defining patient-centered procedures to monitor, compare, and enhance the care provided to patients with macular degeneration (18).

## Implementing patient-centered care in ophthalmology

Ophthalmology practitioners have explored diverse methods to advance patient-centered care, particularly in the treatment of chronic eye diseases. A limited number of studies have been carried out, aimed at specific eye conditions (16,17) or visual impairment overall (19). However, there is still a lack of a holistic patient-centered approach that ophthalmologists could integrate into their practice. Essential goals in the management of chronic eye conditions are improving the understanding of diseases, promoting effective communication between physicians and patients, and addressing the mental well-being of individuals (10).

To date, research on patient-centered care in ophthalmology has been predominantly directed toward the management of glaucoma. This emphasis is attributed to the nature of glaucoma, which often manifests as an irreversible and vision-threatening condition demanding extended patient commitment to controlling IOP. Several studies have raised concerns regarding the existing treatment approaches and outcomes associated with glaucoma (12,14,20,21). The primary challenge is that, akin to other chronic diseases, individuals with glaucoma typically display minimal symptoms in the early stages of the condition, when their visual function remains unchanged. Consequently, during this initial phase, patients are not aware of the severity of the disease, which may lead to reduced motivation for consistent follow-up and treatment adherence. The inadequate perception is further exacerbated by patients’ lack of knowledge about the disease (10,12,14,20-22).

This assertion is substantiated by several studies indicating a lack of awareness regarding glaucoma both within the general population and among individuals diagnosed with the condition (20-22). Notably, 13% of glaucoma patients were unaware of the preventive effects of antiglaucoma drugs on blindness and merely 25.8% of patients could recall the specific eye drops they used (20,21). Due to a limited understanding of the disease, individuals with glaucoma may adhere to their treatment plan without comprehending how it contributes to slowing the progression of the condition. Given the chronic nature of glaucoma and the potential lack of awareness regarding the importance of disease management, patients may not consistently comply with medication and follow-up advice (10). Moreover, physicians and patients diverge in expectations concerning glaucoma treatment. Overall, 10% of patients believe they should play a more active role in decision-making regarding glaucoma treatment, a viewpoint not shared by any ophthalmologists surveyed (23).

Patients frequently rely on the information provided by their treating ophthalmologist. Simultaneously, there is a prevailing belief among many ophthalmologists that patients should receive at least some of their glaucoma education beyond the clinical setting. The limited engagement of patients in decision-making about their treatment may lead to loss to follow-up and non-adherence to taking medication (24,25).

To date, various patient-centered approaches to glaucoma care have been explored, including comprehensive glaucoma education, patient-centered communication strategies during consultations, personal glaucoma records, and joint medical examinations. All methods, except for the glaucoma personal record, have improved patients’ understanding of glaucoma, enhanced the satisfaction of patients, facilitated a more comprehensive grasp of the patient's situation, and lowered the levels of stress or anxiety in both patients and staff (10,12,23,24,26). Moreover, a recent study identified a noteworthy association between high anxiety in glaucoma patients and peak IOP, disc atrophy, and retinal nerve fiber thickness (26). The implementation of patient-centered care may alleviate anxiety since patients feel more empowered to manage their disease and treatment.

Other chronic eye diseases, such as cataracts, are often treated with simple surgery. In these diseases, the outcomes depend less on patient adherence and may not require active patient involvement in treatment. This could explain the limited interest in research in these areas. In terms of care for the visually impaired, on-site eye care has proven effective in improving the vision of residents in care facilities (10). Furthermore, educational programs for caregivers have significantly increased their knowledge, which aligns with the outcomes observed in patient education for glaucoma care (8,10,24).

## Future perspectives

The person-centered approach emphasizes clinicians actively involving patients in managing their health conditions. Ophthalmologists are encouraged to adopt a patient-centered, rather than a physician-centered, approach to consultations. Healthcare providers should make dedicated efforts to educate patients, particularly about medication and surgical procedures, aiming to dispel misconceptions through methods like patient-nurse counseling or group discussions among patients. Additionally, telemedicine is emerging as a potential breakthrough in delivering patient-centered care, bringing increased cost-effectiveness and enhanced patient satisfaction (8,27).

Individuals with significant visual impairment often depend heavily on caregivers for tasks such as administering eye drops and accompanying them to appointments. Unfortunately, existing research has not sufficiently addressed the extent of caregivers' needs. There is a noticeable gap in studies focusing on understanding and supporting the challenges faced by caregivers. To address this gap, caregivers should be equipped with comprehensive information and guidance on providing optimal patient care. This proactive approach aims to ensure a smooth transition from clinical settings to the home environment (10).

Despite the considerable potential of patient-centered care to enhance patients’ well-being, its practical application encounters significant limitations. The primary challenge arises from the restricted time available during consultations. Substantial research underscores the need for extended consultation times to facilitate patient-centered communication and improve aspects related to the vision-related quality of life (25,28). When actively engaging in patient-oriented communication, ophthalmologists on average devoted an additional 1.9 minutes to consultations. This poses a considerable challenge given the time limitation allocated for consultation, particularly in the context of public health services (25). Operating within time constraints, ophthalmologists predominantly prioritize clinical parameters, using them as objective benchmarks for monitoring evidence-based practices, adjusting treatment plans, and evaluating outcomes. Consequently, they may lack the additional time required for patient education regarding the illness, discussing treatment plans, or evaluating the subjective experience of patients. Nevertheless, with increased practice, clinicians may integrate patient-centered communication even within these time limitations. Despite this, allocating an additional 1.9 minutes may prove beneficial if it contributes to improvement in the patients’ overall well-being (10,25).

Furthermore, patients’ better understanding of their disease acquired through pre-visit education can rationalize the time the physician spends addressing questions during the consultation. Additional challenges include the shortage of educators and financial constraints, as the implementation of educational programs or individual counseling services would incur extra costs. Addressing this issue requires the involvement of health policymakers. More resources should be allocated to enhance the quality of care. Personalized patient education, which, despite being expensive and time-consuming, has led to improvements in knowledge, mental well-being, and medication adherence (12,25,29). However, the financial costs of personalized education and time invested in improving clinical outcomes remain unexplored (10,29,30).

Conducting patient education could be as straightforward as offering informational pamphlets or self-study materials available online, thus reaching a large group of patients simultaneously. An ophthalmologist need not be the exclusive provider of patient-centered care. Trained staff members can contribute to patient education, offering a cost-effective method for patients to express their concerns. In this way, the entire medical team is collaboratively working to enhance disease management, rather than placing sole responsibility on clinicians. For instance, assistants can provide additional education to patients after reviewing an information leaflet or website, thereby maximizing efficiency in both time and resources. The cost-effectiveness of patient education can be further enhanced by group education and knowledge sharing. Considering the current trend of virtual communication that began during the COVID-19 pandemic, it is worth exploring the option of group patient education through virtual meetings. Additionally, recognizing the patient's cultural background is crucial when integrating patient-centered care. Hence, clinicians should strive to establish an environment that encourages patients to openly express their perspectives on disease management (10,29,31,32).

Patient-centered care may increase patient awareness regarding the disease, alleviate patients’ anxiety, and improve their understanding and satisfaction (31,32). Unfortunately, there is insufficient research regarding patient-centered care in ophthalmology, making it challenging to determine the extent to which clinical parameters can be improved and consequently impact disease outcomes in this field. Given the value of promoting collaborative care between health care providers and patients, it is important to encourage ophthalmologists to adopt a patient-centered approach. This will enable ophthalmology centers around the world to study the integration of patient-centered care as a routine aspect of medical management (3,5,10).

Current health care models are inadequately suited for the future, proving to be economically and humanistically unsustainable. Consequently, there is a compelling demand, increasingly expressed by patients themselves, for a transition from impersonal, fragmented, and decontextualized health care systems to more personalized, integrated, and contextualized models of clinical practice (3-5).

To address the ongoing crisis in medicine, encompassing challenges in knowledge, care, compassion, and cost, there is a need to prioritize methods that facilitate inexpensive biomedical and technological advancements. This progress should be achieved within a human-centered form of care, recognizing the significance of using scientific knowledge in a way that honors the patient as a holistic individual. This approach involves considering patients’ values, choices, ambitions, cultural background, fears, concerns, and expectations. It acknowledges and addresses their social, spiritual, and emotional needs, as well as their physical requirements. Such an approach is embodied in person-centered medicine, representing a contemporary form of clinical practice (2,3).
